# Complete mitochondrial genome of the Salangid icefish *Neosalanx taihuensis* (Actinopterygii: Osmeriformes: Salangidae)

**DOI:** 10.1080/23802359.2018.1511840

**Published:** 2018-10-30

**Authors:** Ying Wang, Hanyu Wen, Jiyuan Yao, Kaixun Sun, Wenbo Wang, Hongyan Liu, Dong Yang, Fanrong Zhang, Fei Xiong

**Affiliations:** College of Life Sciences, Jianghan University, Wuhan, PR China

**Keywords:** *Neosalanx taihuensis*, mitochondrial genome, phylogenetic analysis

## Abstract

The complete mitochondrial DNA genome of the Salangid icefish (*Neosalanx taihuensis*) was sequenced by the primer walking sequence method. The entire mitochondrial genome of this species is 17,035 bp in length, making it the longest among the reported mitochondrial genomes of *Osmeriformes*. It contains 13 protein-coding genes, 2 ribosomal RNA (rRNA) genes, 22 transfer RNA (tRNA) genes, and one control region (CR). The gene arrangement, nucleotide composition, and codon usage pattern of the mitochondrial genome are similar to those of other teleosts except for two long tandem repeats in the CR. A 486 bp tandem repeat fragment was identified that comprises 2 copies of 243 bp motif and accounts approximately 35.5% of the CR. The 243 bp tandem repeat motif can be folded into a stem-loop secondary structure. Phylogenetic analysis based on 12 concatenated protein-coding genes of the heavy strand shows the genus *Neosalanx* diverged most recently and clustered with *Protosalanx hyalocranius* as a clade.

*Neosalanx taihuensis* belongs to the genus *Neosalanx* within the family *Salangidae* of the order *Osmeriformes*, which is an economically important fish. This species is endemic to China and inhabits mainly the middle and lower reaches of the Yangtze River, including its tributaries and affiliated lakes (Froese and Pauly 2017) . In recent years, because of water pollution, overfishing and habitat destruction, the population of *N. taihuensis* has undergone the loss of appropriate habitat and the decrease in population size (Li 2001; Wang et al. 2005). Therefore, the genetic information of *N. taihuensis* is very urgent to demand.

The fish was obtained from the Taihu Lake (120°19′E, 31°25′N), Jiangsu Province in 2012. After morphological identification, the specimen was deposited in our laboratory (Animal genetics lab, Jianghan University). The fin clips were sampled and then preserved in 95% ethanol. Total genomic DNA was extracted using standard phenol-chloroform methods (Sambrook and Russell 2001). Seven sets of primers were designed from the conservative regions based on the alignment of complete mitochondrial genomes available within the family *Salangidae*.

The complete mitochondrial genome sequence of *N. taihuensis* was 17,035 bp in length (GenBank Accession number MH348204). The protein-coding genes, rRNA genes, tRNA genes, and one control region (CR) of the mitochondrial genome were annotated using MitoAnnotator (Iwasaki et al. 2013). The mitochondrial genome of *N. taihuensis* consisted of 13 typical vertebrate protein-coding genes, 22 tRNAs, 2 rRNAs, and a CR. The gene order and gene content of the mitochondrial genome of *N. taihuensis* are similar to those of other teleosts (Broughton et al. 2001; Zhong et al. 2015). Among the 13 protein-coding genes, ten mitochondrial genes (ND1, ND2, COX1, ATP8, ATP6, COX3, ND3, ND4L, ND5, and ND6) are encoded by the typical TAA or TAG stop codons, while the remaining three mitochondrial genes are encoded by an incomplete stop codon T(aa). The CR is a 1369 bp sequence with two long tandem repeats located between the tRNA-Pro and tRNA-Phe genes, which is the longest among the CR reported of the family Salangidae. The tandem repeat motif is 243 bp long, which can be folded into a stable stem-loop secondary structure.

To explore the evolutionary status of *N. taihuensis* within the order Osmeriformes, a maximum likelihood (ML) tree was constructed using RAxML version 8.1.17 provided by Heidelberg Institute for Theoretical Studies, Heidelberg, Germany (Stamatakis 2014). Phylogenetic tree showed that the genus *Neosalanx* diverged most recently and clustered with *Protosalanx hyalocranius* as a clade (Figure 1). In conclusion, the information of *N. taihuensi* mitochondrial genome reported here may facilitate further investigations of molecular evolution of species in the order Osmeriformes.

**Figure 1.**
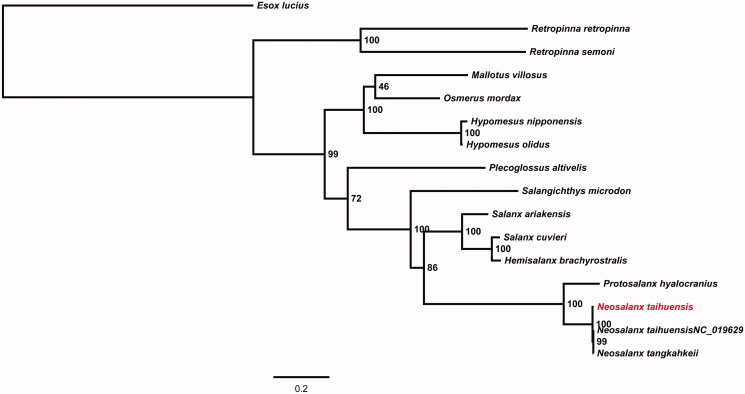
Phylogenetic tree of Osmeriformes based on the Maximum Likelihood (ML) analysis of 12 concatenated mitochondrial protein-coding genes (with the exception of ND6). The bootstrap values for the ML analysis are shown on the nodes. Note: The red taxon represents the species in this study.
